# Management of cerebrospinal fluid leak after anterior clinoidectomy through an endoscopic endonasal approach: report of two cases

**DOI:** 10.1016/j.bjorl.2026.101846

**Published:** 2026-05-29

**Authors:** Rosario María Pérez Contreras, Melisa Rocío Gerloff, Enrique Herrera, Oscar Alejandro Paoletti

**Affiliations:** a Sanatorio Allende, Department of Otolaryngology, Córdoba, Argentina; b Sanatorio Allende, Department of Neurosurgery, Córdoba, Argentina

## Abstract

• Endoscopic endonasal repair successfully managed CSF leaks after clinoidectomy.
• Multilayer reconstruction achieved durable, recurrence-free closure.
• Gasket-seal and nasoseptal flap techniques provided watertight repair.
• Technique selection should be individualized according to defect features.

## Introduction

The management of Cerebrospinal Fluid (CSF) leak following anterior clinoidectomy represents a challenging complication, typically unresponsive to conservative treatment.

Anatomically, the Anterior Clinoid Process (ACP) is a bony structure located on the medial surface, along the posterior margin of the lesser wing of the sphenoid bone. Its anterior root forms the roof of the optic canal, while the posterior root separates the superior orbital fissure from the optic canal. Anterior clinoidectomy has several indications within the field of neurosurgery.

In cases where pneumatization of the posterior root is present (reported in 4%–29.3% of cases), anterior clinoidectomy creates a communication between the subarachnoid space and the sphenoid sinus, making defect closure particularly difficult.^1^

The overall frequency of CSF leaks in elective cranial surgeries is approximately 3.8%. Skull base procedures carry the highest rate of postoperative CSF leak, reaching 6.2%.^2^ When CSF leakage occurs and conservative management fails, surgical repair is required.

Traditional repair can be achieved via craniotomy and direct defect packing.^3^ Alternatively, endoscopic endonasal repair has emerged as a valuable option, allowing direct visualization and closure of the defect without the need for craniotomy. This approach provides excellent visualization of the surgical field. Published series have demonstrated that it is both safe and effective, with success rates exceeding 96%, low recurrence of CSF leak (3.8%), and minimal complications in cohorts of up to 263 patients.^4^

The techniques and materials used for defect closure vary widely, but no significant difference in success rates has been demonstrated among the different graft types.

A “double-layer” technique may be used, consisting of fat or muscle in an inlay position, covered by fascia or another graft, as described in studies from 2016 to 2025.^1^^,^^4^ Pedicled septal flaps are frequently used in high-flow leaks, often combined with a fat graft.^1^

Finally, multilayer closures may be performed using both soft and rigid grafts to achieve a watertight “gasket seal” effect,^5^ subsequently reinforced with a vascularized flap.

We herein present our experience with two patients: one treated using the gasket-seal technique and the other with a multilayer nasoseptal flap reconstruction.

## Case report

### Case 1

A 64-year-old female patient presented to the Department of Neurosurgery in 2020 with a diagnosis of a right anterior clinoid meningioma and a carotid-hypophyseal aneurysm. She underwent surgery via a pterional approach with extradural anterior clinoidectomy. Tumor resection and aneurysm clipping were successfully performed. The reconstruction phase began with a watertight dural closure using 4‒0 Prolene sutures, reinforced with 3 mL of fibrin glue and dural stay sutures at the craniotomy margins. The bone flap was repositioned and secured with 2‒0 silk sutures. A cranioplasty was performed using surgical cement (Subiton). A multi-layer closure was executed, including the galea with 3‒0 Vicryl and a continuous subcuticular suture with 3‒0 Monocryl for the skin.

**Fig. 1:**
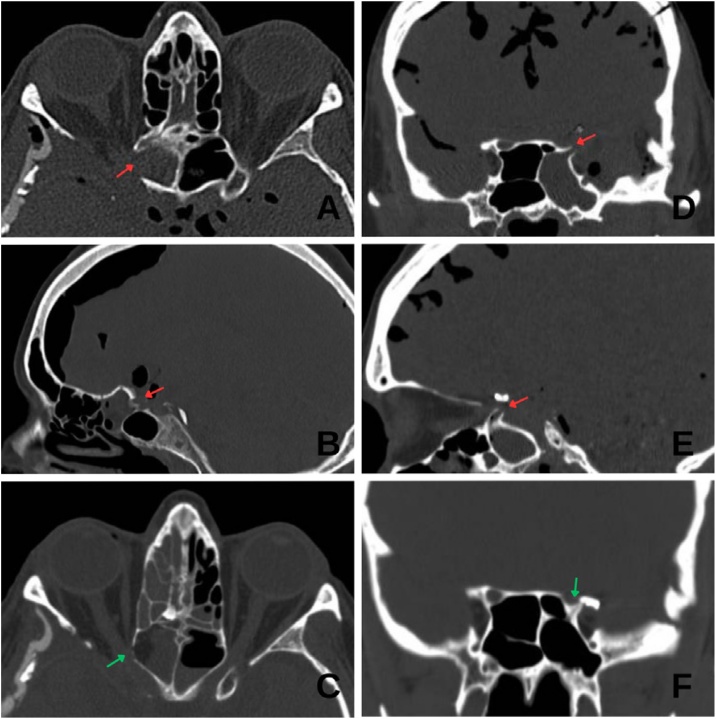
Pre- and postoperative CT scans of Cases 1 and 2. (A and B) Axial and sagittal preoperative views of Case 1 showing marked pneumocephalus and the defect area (red arrow). (C) Postoperative axial view of Case 1 showing resolution of the defect (green arrow). (D and E) Coronal and sagittal preoperative views of Case 2 showing pneumocephalus and the defect (red arrow). (F) Postoperative coronal view showing resolution of the defect and absence of pneumocephalus (green arrow).

On postoperative day 4, the patient developed COVID-19 pneumonia. She was isolated, and all protective measures were taken. On postoperative day 9, she developed CSF rhinorrhea from the right nostril. A Computed Tomography (CT) scan revealed a skull base defect with right predominant pneumocephalus (Fig. 1), without signs of intracranial hypertension. Conservative management with acetazolamide and bed rest was initiated.

After seven days of conservative treatment and resolution of COVID-19, persistent CSF rhinorrhea upon mobilization was noted despite lumbar drainage. Endoscopic endonasal surgery was therefore indicated. Multilayer closure was performed using fat within the defect, fascia lata, and a nasoseptal mucosal flap. The defect was repaired (Fig. 1), CSF leak did not recur, and the patient was discharged seven days after surgery.

### Case 2

A 40-year-old female patient presented with a 6 mm left carotid-ophthalmic aneurysm. She underwent elective surgery. An extradural approach was used to identify the orbitomeningeal band and the sphenoid wing. An extradural anterior clinoidectomy was performed, followed by optic canal unroofing to improve the exposure. After a C-shaped dural opening and Sylvian fissure dissection, the superior-medially projected aneurysm was identified. Proximal control was maintained via a temporary clip on the cervical common carotid artery for 2 min, allowing for definitive clipping of the aneurysm with a straight clip while preserving the local vascular anatomy. The dural closure was performed in a watertight fashion, reinforced with 3 mL of biological adhesive after the placement of a subdural ICP monitor. The bone flap was repositioned and secured using a titanium fixation system consisting of one mesh and four plates (three 5-hole and one 6-hole plates) with six titanium screws. The soft tissues were closed in layers according to standard surgical technique.

On postoperative day 4, she developed CSF rhinorrhea through the left nostril, associated with headache and mild speech disturbances. Conservative measures were initiated, including bed rest and acetazolamide. A CT scan (Fig. 1) showed extensive pneumocephalus and a skull base discontinuity at the level of the anterior clinoid process, involving the superior-lateral wall adjacent to the cavernous sinus and near the surgical clip.

**Fig. 2:**
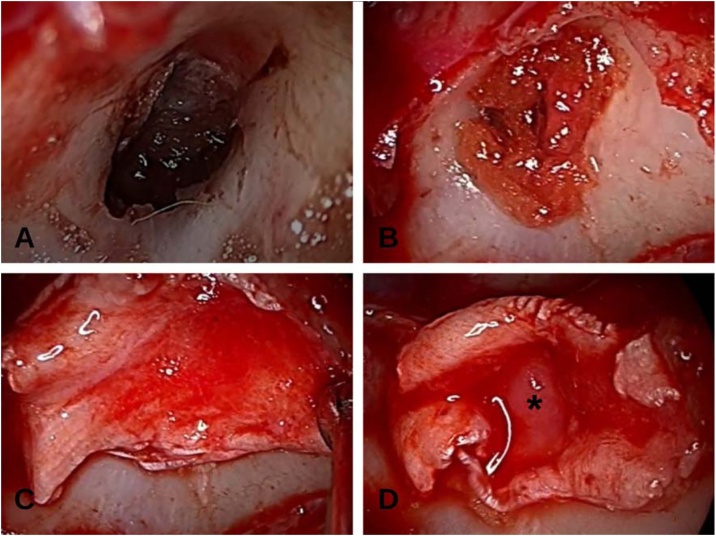
“Gasket seal” technique for management of CSF fistula following anterior clinoidectomy. (A) Defect after anterior clinoidectomy. (B) Fat graft. (C) Fascia lata. (D) Placement of septal cartilage over the fascia lata in a plug-like configuration covering the defect. (*) Septal cartilage.

An endoscopic endonasal approach was planned. Upon entering the sphenoid sinus, a skull base defect was identified over the left opticocarotid recess (Fig. 2). It was repaired using a multilayer technique: fat packing within the defect, overlaid with abdominal fat and fascia lata, and then covered with a septal cartilage graft to create a gasket-seal effect. The defect was successfully repaired (Fig. 1). The patient was discharged seven days after surgery with no recurrence of CSF leakage.

## Discussion

As clearly stated by Nyquist et al. in their 2011 study,^6^ “The CSF leak cannot be repaired if it cannot be identified”. The principal advantage of the endoscopic approach lies in its superior visualization. In both our cases, an endoscopic approach allowed rapid identification of the defect without the need for craniotomy. Previous studies, such as that of Nasirmohtaram et al.,^4^ support the endoscopic approach with success rates of up to 96.2%. Although their case series focused on spontaneous or traumatic fistulas, their results support the use of multilayer techniques as well as the endoscopic approach.

In addition to endoscopic repair, several transcranial neurosurgical strategies have been described for both prevention and treatment of CSF leaks after anterior clinoidectomy. Traditional management includes reopening the previous craniotomy with direct packing of the skull base defect, which allows direct access to the clinoid region but requires repeat intracranial manipulation. Preventive techniques have also been proposed, such as the “yo-yo” technique described by Chi et al., in which a muscle graft is secured over the pneumatized optic strut at the time of aneurysm clipping.^7^ More recent neurosurgical series have emphasized meticulous transcranial reconstruction of the clinoid defect, using a hybrid techinque, as an alternative to endonasal repair.^8^ While these strategies remain valid, the endoscopic endonasal approach offers excellent visualization of the defect and avoids repeat craniotomy.

Preoperative (pre-clinoidectomy) CT imaging suitable for classification of ACP pneumatization was not available in either case. Therefore, a formal CT-based anatomical classification could not be assigned. This limitation underscores the importance of preoperative high-resolution CT assessment of anterior clinoid pneumatization, which may help anticipate the risk of sinonasal communication and postoperative CSF leakage after clinoidectomy.^9^

In both patients, multilayer techniques were used. In Case 1, the defect was repaired using a fat plug, fascia, and a nasoseptal mucosal flap. The nasoseptal flap is well supported in the literature for the treatment of high-flow CSF leaks or large skull base defects. Beer-Furlan et al.^1^ propose a stepwise, graded approach to reconstruction.

In Case 2, the “gasket seal” method (Fig. 1) described by Leng et al.^5^ and supported by Nyquist et al.^6^ was used with excellent results. This technique is based on the compression of a soft graft against the defect using a rigid buttress, thus achieving a watertight closure. It is particularly effective when the defect has a bony rim that allows for firm fixation of the graft. In our patient, fascia lata was placed over the defect and compression was achieved with a septal cartilage graft.

Key technical pearls for the “gasket seal” method include achieving a wide exposure of the bony margins combined with careful denudation of the surrounding mucosa, which is essential to promote optimal graft adherence. Proper sizing of the graft is crucial; it must ensure sufficient overlap beyond the defect margins to prevent displacement. During placement, achieving firm positioning of the graft and buttress is fundamental to obtain a watertight seal.

Similar results have been reported by Shaftel et al.^10^ who presented a series of four patients with CSF rhinorrhea after anterior clinoidectomy who were treated through an endoscopic transsphenoidal approach. Reconstruction was performed using a free nasal mucosal graft, and no recurrences were observed during a mean follow-up of 12.5 months. While their repair strategy was based on free grafts, our experience suggests that multilayer techniques incorporating vascularized flaps or a gasket-seal technique may offer added stability.

Both patients reported satisfaction with the surgical management and postoperative care. Clinical follow-up showed an uneventful course, with no signs of meningitis, persistent headache, or recurrence of the fistula, consistent with what has been reported in the literature for low- to moderate-flow leaks, where a well-executed anatomical closure is often sufficient.^5^^,^^10^

## Conclusion

The endoscopic approach is an effective option for the treatment of cerebrospinal fluid leaks secondary to anterior clinoidectomy. The choice of surgical technique for defect closure should be tailored to each individual patient.

## ORCID ID

Rosario María Pérez Contreras: 0009-0006-5238-4714

Melisa Rocío Gerloff: 0009-0007-2114-3384

Enrique Herrera: 0009-0002-8661-3811

Oscar Alejandro Paoletti: 0009-0003-5643-3294

## Ethical considerations

Authorization was obtained from the Institutional Ethics Committee (approval dated September 18, 2025).

## Consent to participate

Written informed consent was obtained from all patients for publication of this case report and accompanying images, in accordance with institutional guidelines.

## Patient consent

Written informed consent was obtained from the patients for publication of this case report and accompanying images. A copy of the written consent is available for review by the Editor-in-Chief of this journal on request.

## Funding

The author(s) received no financial support for the research, authorship, and/or publication of this article.

## Data availability

The authors declare that all data are available in repository.

## Declaration of competing interest

The authors declare no conflicts of interest.
